# *Carthamus tinctorius* L. (Safflower) Flower Extract Attenuates Hepatic Injury and Steatosis in a Rat Model of Type 2 Diabetes Mellitus via Nrf2-Dependent Hypoglycemic, Antioxidant, and Hypolipidemic Effects

**DOI:** 10.3390/antiox13091098

**Published:** 2024-09-10

**Authors:** Nuha Saad Alshareef, Sahar Abdulaziz AlSedairy, Laila Naif Al-Harbi, Ghedeir M. Alshammari, Mohammed Abdo Yahya

**Affiliations:** Department of Food Science and Nutrition, College of Food Science and Agriculture, King Saud University, Riyadh 11451, Saudi Arabia; 439204479@student.ksu.edu.sa (N.S.A.); ssudairy@ksu.edu.sa (S.A.A.); aghedeir@ksu.edu.sa (G.M.A.); mabdo@ksu.edu.sa (M.A.Y.)

**Keywords:** *Carthamus tinctorius*, safflower, liver, NAFLD, oxidative stress, Nrf2

## Abstract

This study aimed to examine the hepatic and anti-steatotic protective effects of methanolic extract from *Carthamus tinctorius* (safflower) flowers (SFFE), using a rat model of type 2 diabetes mellitus (T2DM), and to examine the molecular mechanisms underlying these effects. Adult male Wistar rats were used for this study. First, T2DM was induced in some rats by feeding them a high-fat diet (HFD) for 4 weeks, followed by a single dose of streptozotocin (STZ) (35 mg/kg, i.p.). Experimental groups included the following five groups (n = 8 in each): control, control + SFFE, T2DM, T2DM + SFFE, and T2DM + SFFE + brusatol (an Nrf2 inhibitor, 2 mg/kg, i.p.). SFFE was administered at a concentration of 300 mg/kg, and all experiments concluded after 8 weeks. Treatments with SFFE significantly reduced fasting blood glucose levels, free fatty acids (FFAs), cholesterol, triglycerides, and low-density lipoprotein cholesterol in both the control and T2DM rats, but they failed to reduce fasting insulin levels in these groups. SFFE treatments also improved the liver structure and reduced hepatocyte vacuolization and hepatic levels of triglycerides and cholesterol in T2DM rats, in addition to increasing the hepatic mRNA levels of keap1 and the cytoplasmic levels and nuclear activities of Nrf2 in both the control and T2DM rats. SFFE also stimulated the expression levels of PPARα and CPT-1 but reduced the malondialdehyde (MDA), mRNA levels of SREBP1, fatty acid synthase, and acetyl CoA carboxylase in both the control and T2DM rats; meanwhile, it reduced hepatic mRNA and the nuclear activities of NF-κB and increased levels of glutathione, superoxide dismutase, and heme oxygenase-1 in the livers of both groups of treated rats. Furthermore, SFFE suppressed the levels of caspase-3, Bax, tumor necrosis factor-α, and interleukin-6 in the T2DM rats. Treatment with brusatol prevented all of these effects of SFFE. In conclusion, SFFE suppresses liver damage and hepatic steatosis in T2DM through Nrf2-dependent hypoglycemic, antioxidant, anti-inflammatory, and hypolipidemic effects.

## 1. Introduction

Non-alcoholic fatty liver disease (NAFLD) is considered the most common and significant global hepatic disease that is positively associated with type 2 diabetes mellitus (T2DM) and insulin resistance (IR) [1]. The disease has different spectra, which can start as simple steatosis but later develop into more advanced stages, such as steatohepatitis, hepatic fibrosis, and hepatic failure [1]. Recent reports have regarded NAFLD as a silent epidemic that should draw significant attention toward finding suitable prevention strategies and treatments [2]. Therefore, understanding the molecular basis of the etiology of the disease is a key factor in finding such therapies.

To date, the molecular basis underlying NAFLD has been published in numerous reviews [3,4,5], where it is well documented that NAFLD develops due to an imbalance in the equilibrium between hepatic triglyceride (TG) synthesis and cleanup [3]. However, as described in two- and multiple-hit models, oxidative stress and the resulting hepatic inflammation are key events in the progression of the disease [6,7]. Accordingly, the first stage of the disease involves dyslipidemia, increased hepatic lipid synthesis and accumulation as a result of hyperglycemia, an increased influx of free fatty acids (FFAs) from adipose tissue, and impaired hepatic TG excretion and oxidation [7]. The second stage of the disease is triggered mainly by the increased hepatic production of reactive oxygen species (ROS) and inflammatory cytokines, which act in a vicious cycle to induce hepatic inflammation, insulin resistance (IR), and apoptosis [4,6,7]. At the clinical level, mitochondrial dysfunction, increased and impaired FA oxidation, and higher levels of ROS were observed in the liver biopsies of patients with NAFLD [8,9]. In addition, ROS were shown to be the major molecular mechanism that drives hepatic inflammation, fibrosis, and injury in the liver tissues of animals or patients with NAFLD [10,11].

Additionally, NAFLD is associated with the impaired function of transcriptional factors and nuclear receptors, which regulate lipid and glucose metabolism, inflammation, and cellular redox potential [12]. Accumulating data show an emerging role of nuclear factor-erythroid 2-related factor 2 (Nrf2) in the protection against liver disorders, particularly NAFLD [13,14,15,16]. Like in most other cells, the transcription factor Nrf2 is abundantly expressed in the liver cells, where it acts mainly to stimulate the expression levels of glutathione (GSH) and several other antioxidant genes [16]. Additional evidence also indicates an important role of Nrf2 in regulating FA and lipid metabolism in the liver, and Nrf2 deficiency has been reported as a major contributor to the development of hepatic steatosis and NAFLD [13,15,17]. Indeed, mice deficient in Nrf2 and fed either a normal or high-fat diet showed increased hepatic markers of oxidative stress and inflammation and had accelerated lipid deposition in their livers, as compared to wild-type mice under the same feeding conditions [18,19,20,21,22]. Further studies have revealed that Nrf2 is an important regulator that can reduce hepatic FA uptake, inhibit lipogenesis, and stimulate mitochondrial FA by regulating the transcription of several enzymes and proteins involved in these pathways [17,23,24,25,26]. In addition, it has been shown that rodents fed a high-fat diet (HFD) showed reduced expression and activities of Nrf2 that were associated with the severity of liver damage and the degree of hepatic steatosis [27,28,29]. However, overactivation or hepatocyte-specific activation of Nrf2 prevented hepatic steatosis and the progression of nonalcoholic steatohepatitis (NASH) in HFD rats by suppressing hepatocyte oxidative damage, inflammation, and lipid accumulation [17,29,30,31,32,33]. Therefore, researchers are invested in finding safe and non-expensive Nrf2 activators as potential therapeutic options with which to treat NAFLD [34].

Herbal medicines containing natural plant-derived compounds are currently receiving significant attention for the treatment of NAFLD, due to their multiple pharmacological activities [35]. In addition, several plant phytochemicals can prevent and/or treat liver disorders, including NAFLD, by activating the Nrf2/antioxidant pathways [33,36,37]. *Carthamus tinctorius* L. (*C. tinctorius*), commonly known as safflower, is a traditional plant that is cultivated in China, the Middle East, and other areas of the world [38]. The flowers and seeds of this plant have been used in traditional medicine as oil and in meals as a natural colorant and food additive, as well as to treat pain, osteoporosis, swelling, allergies, hypertension, cardiovascular disease, osteoporosis, cancer, inflammatory conditions, and skin disorders, due to its antioxidant, antibacterial, anti-inflammatory, and antitumorigenic effects [39]. The antioxidant and anti-inflammatory potentials of this plant were attributed to its potential to scavenge and reduce the production of ROS, thus suppressing NF-κB and inflammatory cytokine production and stimulating antioxidant expression (e.g., hemeoxygenase-1 (HO-1) and superoxide dismutase (SOD)) [40,41,42,43,44]. In addition, hypoglycemic, insulin-releasing, and hypolipidemic activities of the seed and flower have been reported in rat and mouse models of T1DM, induced by alloxan and streptozotocin (STZ) [45,46,47]. The seeds and flowers also alleviated dyslipidemia and reduced hepatic de novo lipid synthesis by suppressing genes involved in triglyceride (TG) and cholesterol (CJOL) synthesis, while stimulating those related to FA mitochondrial oxidation [48,49,50]. Of interest, the hepatic protective effects of *C. tinctorius* seeds and oil extracts were also demonstrated on some models of liver toxicity, such as carbon tetrachloride (CCl_4_), where protection was associated with reduced ROS generation, thus decreasing the activity of NF-κβ and stimulating the Nrf2 signaling pathway [51,52,53]. This plant was also shown to prevent endothelial inflammation in human umbilical cells via Nrf2/HO-1 axis-mediated reductions in ROS generation and NF-κB activation [54]. These data confirm the Nrf2 stimulatory potential of *C. tinctorius*.

Studies on the effects of seed or oil extracts from safflower on NAFLD are very rare. In a single study, Hu et al. [55] showed that safflower yellow, a major ingredient in the flowers of *C. tinctorius*, alleviated metabolic-associated hepatic steatosis in HFD-fed mice by suppressing hepatic fatty acid synthesis genes and endoplasmic reticulum (ER) stress and modulating gut microbiota. On this note, more than 200 substances were reported to exist in the flowers of these plants, of which at least 20 molecules are known to have potent pharmacological activities [56,57]. These data suggest that the hepatic protective effect that could be afforded by the whole flower extract against the damage and steatosis in HFD-fed rats could be more effective. Therefore, in this study, we first examined the hepatic protective effect of methanol extract from *C. tinctorius* flowers against NAFLD in HFD-fed rats. In addition, and given the roles of the Nrf2 signaling pathway in oxidative stress, inflammation, lipogenesis, and apoptosis, we examined whether this extract may act at the molecular level by targeting this pathway.

## 2. Materials and Methods

### 2.1. Animals

In total, 40 adult male Wistar rats (7 weeks old/110 ± 10 g) were obtained from, cared for by, and maintained in the Animal Unit at King Saud University (KSU), Saudi Arabia. The animals were always kept in a separate room with ambient conditions (temperature 22 ± 1 °C) and day/night cycles (12 h for each). They were always housed in plastic cages at 6 rats/cage, where the enrichment environment was wood shaving and paper wool nesting. Animal care, feeding, treatment, anesthesia, and surgery were conducted under the supervision of in-house veterinarians and were approved by the institution’s Animal Use and Care Committee (IRB # KSU-SE-23-85).

### 2.2. Plant Material and Preparation of the Extract

A whole *Carthamus tinctorius* L. plant was purchased freshly from a farm in Riyadh, Kingdom of Saudi Arabia. The plant was verified by a botanist from the College of Pharmacy at KSU, and a voucher specimen was deposited in their museum. The extraction of safflower flowers was conducted at the pharmacognosy lab of KSU, using the normal manual as described by Jun et al. [41]. In brief, the flowers were separated and washed with distilled water several times. They were then dried in an oven at 40 °C, until most of the water was removed. The dried flowers (2 kg) were then extracted using 6 L of 70% methanol with a Soxhlet apparatus for 24 h. The resultant solution was then dried using a rotatory evaporator. This yielded a total dry powder methanol extract of 200 g, which was then freeze-dried until further use. Once used, the powder was dissolved in normal saline to the desired concentration, which was used in the experimental procedure. This extract was named SFFE.

### 2.3. Gas Chromatography-Mass Spectrometry (GC-MS) Analysis 

The GC-MS analysis of methanol (90%) extracts from safflower flowers was performed on a 5975 gas chromatography device coupled with a mass spectrometer (Agilent, Santa Clara, CA, USA; SEM Ltd., Istanbul, Turkey). For the analysis, the stationary phase was the Innowax FSC column (60 m × 0.25 mm, 0.25 µm film thickness), and the mobile phase was helium (0.8 mL/min). The sample volume (0.1 µL) was injected with a split ratio of 40:1. The oven temperature of the GC was set at 60 °C for 10 min, then increased to 220 °C at a rate of 4 °C/min, held for 10 min, then increased to 240 °C at a rate of 1 °C/min. The injector and the transfer line temperatures were 250 and 280 °C, respectively. The MS detection was carried out at 70 eV with a scan mass range of *m*/*z* 35–450.

### 2.4. Diets

For this study, we used commercially available control diet (D12450B, Research Diets, Brunswick, NJ, USA) and HFD (D12492, Research Diets, Brunswick, NJ, USA). The calorie intake of the control diet is 3.85 kcal/g (16.1 kJ/g), of which the percentages of fats, carbohydrates, and proteins are 10%, 70%, and 20%, respectively. The major ingredients of the control diet (g/kg) were 200 g casein, 50 g cellulose, 350 g sucrose, 315 g corn starch, and 20 g lard. The total energy intake from the D12492 HFD is 5.24 kcal/g (21.9%), obtained from 60% fat, 20% carbohydrates, and 20% protein. The major ingredients of the HFD were 200 g casein, 50 g cellulose, 0.0 g corn starch, 68.8 g sucrose, and 245 g lard.

### 2.5. Establishment of T2DM

A high-fat diet (HFD), in addition to a low intraperitoneal dose of STZ (35 mg/kg), was used to establish T2DM in these rats, a method previously used by other researchers [17,58]. The rats were randomly divided and fed either the control diet or HFD for 4 weeks. At the end of this treatment period, a low dose of STZ (35 mg/kg) dissolved in sodium citrate buffer (pH = 5.5) was intraperitoneally administered to the HFD-fed rats in a final volume of 1 mL. The control rats were administered an equivalent volume of normal saline. Three days later, the blood glucose levels in both groups were measured. The blood glucose levels of the control rats showed normal fasting glucose levels. However, those HFD rats with blood glucose levels higher than 11 mmol/L were considered to have T2DM and were included in further experimentation.

### 2.6. Experimental Design

Control and rats with preestablished T2DM-fed rats were randomly classified into 5 groups (n = 8 rats/group), as follows: 

Group (1) (control group): normal rats that were fed normal diet and orally treated with normal saline as vehicle; Group (2) (control + SFFE group): normal rats that were fed control diet and orally treated with SFFE at a dose of 300 mg/kg; Group (3) (T2DM model rat group): rats with pre-established T2DM that were fed normal diet and were orally treated with normal saline as vehicle; Group (4) (T2DM + SFFE-treated group): rats with pre-established T2DM that were fed normal diet and orally treated with SFEE at a dose of 300 mg/kg; and Group (5) (T2DM + SFFE + brusatol group): rats with pre-established T2DM that were fed normal diet and orally treated with SFEE at a dose of 300 mg/kg and concomitantly received brusatol (a selective Nrf2 inhibitor) at a dose of 2 mg/kg (i.p.). All treatments were administered by gavage and continued for 8 weeks. Body weights were monitored every weekend.

### 2.7. Dose Selection

Brusatol is a potent Nrf2 inhibitor that can be administered in vivo to block the multisystemic activities of Nrf2 [59,60,61]. Brusatol was always administered 1 h before the administration of SFEE. In our preliminary research, we treated the diabetic rats with increasing doses of SFEE (100, 200, and 300 mg/kg) and noticed a dose-response reduction in fasting serum glucose, insulin, TGs, and cholesterol; in addition, none of these doses showed toxicity in control rats. For these reasons, we selected a dose of 300 mg/kg as our therapeutic dose for this study, which was additionally supported by many other studies showing that water, ethanol, and methanol extract at doses between 200 and 500 mg/kg have no signs of toxicity but have favorable effects on reducing fasting hyperglycemia, hyperlipidemia, and hepatic cholesterol synthesis and improving liver architectures in T1DM and under high-calorie diet feeding conditions [45,46,47,49,50].

### 2.8. Collection of Blood Samples and Liver Tissues

On the last day of this study, all animals were fasted for 10 h and then anesthetized using a ketamine/xylazine hydrochloride mixture (80/10 *v*:*v*). Equal blood samples (1 mL each) were directly obtained from the right ventricle into either gel-containing or EDTA-containing tubes for the collection of serum and plasma, respectively. In all cases, blood samples were allowed to settle for 30 min at room temperature and then centrifuged at 500× *g* for 10 min. Serum and plasma samples were aliquoted into Eppendorf tubes, labeled, and maintained at −20 °C for further biochemical analysis. Thereafter, all rats were euthanized via cervical dislocation; their livers were collected, washed with ice-cold normal saline, and cut into smaller parts. All of these tissues were placed in liquid nitrogen and then preserved at −80 °C until use. Some parts were directly fixed in 10% buffered formalin for histological evaluation.

### 2.9. Biochemical Analysis from Serum and Plasma

All of the measurements from the serum and plasma were conducted using rat-specific enzyme-linked immunosorbent assay (ELISA) kits, which evaluate the plasma levels of glucose and insulin (Cat. No. 10009582, Cayman Chemicals, Ann Arbor, MI, USA, and Cat. No. 589501, Dallas, TX, USA). The serum levels of cholesterol (CHOL) were measured using one kit (Cat. NO. ECCH-100, BioAssay Systems, Hayward, CA, USA); ELISA kits obtained from MyBioSource (San Diego, CA, USA) were used to measure the levels of low-density lipoprotein cholesterol (LDL-c), triglycerides (TGs), and free FAs (FFAs) (Cat. No. MBS702165, Cat. No. MBS726298, and Cat. No. MBS014345); another kit (Cat. No. ab65337, Abcam, Cambridge, UK) was used to measure the serum levels of glycerol; the serum levels of alanine aminotransferase (ALT), gamma-glutamyl transpeptidase (GGT), and aspartate aminotransferase (AST) were also measured using ELISA kits (Cat. No. MBS269614, MyBioSource, San Diego, CA, USA; Cat. No. MBS9343646, MyBioSource, San Diego, CA, USA, and Cat. No. CSB-E13023r-1, Cosmo Bio, Carlsbad, CA, USA). All measurements were performed in duplicate and for n = 8 samples/group, as per the instructions of each kit.

### 2.10. Measurements of Hepatic Lipids

The extraction of the lipid fractions from the frozen livers followed the guidelines routinely utilized in our labs [61,62], as per the method of Folch et al. [63]. Briefly, each 100 mg of the liver tissues was soaked in equal volumes of a methanol/chloroform mixture and water (0.25 mL each) for 4 h. The lower organic layer was separated after centrifugation (2200× *g*/15 min) and was then evaporated and dried under pressure. The resultant pellet was weighted and reconstituted in isopropanol for further evaluation. The measurements of hepatic TGs, CHOL, and FFAs were performed, using the same aforementioned ELISA kits used to measure their levels in the serum, for n = 8 samples/group.

### 2.11. Measurements of Hepatic Enzymes of Glucose Metabolism

Total homogenates in the liver were prepared by digesting the liver sample (50 mg) in 450 μL of ice-cold phosphate-buffered saline, followed by centrifugation at 4 °C (11,200× *g*). Rat-specific ELISA kits were used to measure the concentrations of glucose-6-phosphatase (6-Pase), (Cat. No. MBS097902, MyBioSource, San Diego, CA, USA), glucokinase (Cat. No. MBS453149, MyBioSource, San Diego, CA, USA), and fructose-1,6-bisphosphatase 1 (PBP1) (cat. No. MBS7227331, MyBioSource, San Diego, CA, USA). All measurements were done in duplicate for n = 8 rats/group.

### 2.12. Measurement of Hepatic Markers of Oxidative Stress, Inflammation, and Apoptosis

ELISA kits were used to measure the levels of necrosis factor-α (TNF-α) and interleukin-6 (IL-6) (Cat. No. ab100785, Abcam, Cambridge, UK, and Cat. No. R6000B, R&D System, Minneapolis, MN, USA). ELISA kits purchased from AFG Scientific (Northbrook, IL, USA) were used to measure the hepatic homogenate levels of malondialdehyde (MDA) (Cat. No. EK720188), total glutathione (GSH) (Cat. No. EK720816), Hemeoxygenase-1 (HO-1) (Cat. No. EK720658), and superoxide dismutase (SOD) (Cat. No. EK720889). The total cytoplasmic activity of Nrf2 was determined using a special ELISA kit (MBS752046, MyBiosource, San Diego, CA, USA).

The total levels of advanced glycation end products (AGEs) were measured using the CusaBio ELISA kit (Cat. No. CSB-E09413r, Houston, TX, USA). Additional ELISA kits were used to measure the total levels of Bax (Cat. No. E4513, BioVision, Milpitas, CA, USA), Bcl2 (Cat. No. LS-F11016, LS Bio, Shirley, MA, USA), and caspase-3 (Cat. No. LS-F4135, LS Bio, MA, USA). All analyses were performed, as per each manufacturer’s instructions, for n = 8 samples/group.

### 2.13. Analysis of the Cytoplasmic and Nuclear Activities of Selected Transcriptional Factors

The nuclear proteins were prepared using tissue-specific cytoplasmic and nuclear fraction isolation kits (# 50296 Active Motif, Carlsbad, CA, USA). The activities of Nrf2, NF-κB, and SREBP1 in the nuclear extracts were analyzed using rat-specific kits obtained from Abcam (Cambridge, UK) (Cat. No ab2072223, Cat. No. Cat. No. Ab133112, and Cat. No. ab133125 Abcam, Cambridge, UK), as per the manufacturer’s instructions, for n = 8 samples/group.

### 2.14. Real-Time PCR of Adipose and Hepatic Tissues

qPCR was used to evaluate the mRNA expression levels of keap1, Nrf2, NF-κB, SREBP1, fatty acid synthase (FAS), acetyl CoA carboxylase (ACC1), carnitine palmitoyltransferase 1 (CPT1), PPARα, and β-actin (a reference gene). Primer gene number, forward and reverse sequences, and amplification size were described in our previous study [29]. RNA was isolated from the frozen liver samples and was used to synthesize the first strand of cDNA using RNassy Mini and cDNA synthetizing kits (Cat. No. 74104, Qiagen; and No. K1621, Thermo Fisher kit, respectively). All amplification reactions were conducted using the SsoFast EvaGreen Supermix kit (Cat. No. 172-5200, Bio-Rad, Hercules, CA, USA) in a CFX96 real-time PCR machine (Bio-Rad, Hercules, CA, USA), as per the manufacturer’s instructions, and as described previously [29]. The transcription levels of each target were presented as normalized to β-actin. All amplification reactions were performed as n = 6 samples/group.

### 2.15. Hematoxylin and Eosin Staining

Liver tissue samples were fixed for 24 h in 10% buffered formalin. Tissues were then treated with ethanol, embedded in paraffin, and cut using a microtome into sections of 3–5 μm. The sections were then routinely H&E stained, examined, and photographed under a light microscope.

### 2.16. Statistical Analysis

Data from all parameters were collected and analyzed with the help of GraphPad Prism analysis software (version 8, USA). All parameters were analyzed using a 2-way ANOVA and a post hoc Tukey’s test. Data were considered significantly different at *p* < 0.05 and are presented or graphed as the mean ± standard deviation (SD).

## 3. Results

### 3.1. GC-MS Composition of Safflower Flowers

The GC-MS chromatogram (Figure S1 and Table S1) of the safflower flower methanol extract revealed 38 compounds (Table S1). The predominant compounds observed in the methanol extract were 6-(3,5-dimethyl-1H-pyrazol-1-yl)-3-methyl-1,2,4-triazolo [4,3-b][1,2,5,6]tetrazine (34.92% of the total area), followed by nonacosane (11.17%), 8-hydroxy-3,8a-dimethyl-5-methylene-2-oxododecahydronaphtho [2,3-b]furan-4-yl acetate (6.58%), hexadecanoic acid, trimethylsilyl ester (5.43%), octadecane (4.04%), anhydrocumanin (3.35%), Cedran-diol, 8S,13-(2.65%), palmitic acid (2.56%), and bicyclo [4.4.0]dec-1-ene, 2-isopropyl-5-methyl-9-methylene-(2.08%). Nonacosane (11.17), obtained from *Baphia massaiensis*, demonstrates little activity against *Escherichia coli*, *Bacillus subtilis*, *Pseudomonas aeruginosa*, and *Staphylococcus aureus*.

### 3.2. SFFE Improves Body and Liver Weights, But Not Food Intake, in T2DM Rats

The final body weights, the gain in body weights, liver weights, liver index, and food intake were not significantly altered between the control and control + SFFE-treated rats (Table 1). The T2DM rats showed a significant increase in their final weekly food intake during the last 4 weeks of this study, as well as in final liver weight and liver index, as compared to the control and control + SFFE-treated rats. T2DM rats also had a significantly lower final body weight and weight gain than control rats (Table 1). Food intake did not significantly change among the T2DM, T2DM + SFFE-, and T2DM + SFFE + brusatol-treated rats (Table 1). On the other hand, there was a significant increase in final body weight and weight gain that was concomitant with a significant reduction in the final liver weight and liver index in the T2DM + SFFE-treated rats, when compared to the T2DM rats (Table 1). The alterations in final body weight, weight gain, final liver weight, and liver index were reversed in the T2DM + SFFE + brusatol-treated rats, whose levels were not significantly different from those observed in the T2DM model rats, as compared with their T2DM + SFFE-treated counterparts (Table 1).

### 3.3. SFFE Attenuates Fasting Hyperglycemia and Inhibits Hepatic Gluconeogenesis in Both Control and T2DM Rats

Fasting levels of glucose and HBA1c%, as well as hepatic levels of key gluconeogenesis enzymes, including G6Pase and FBP-1, were significantly elevated, while fasting insulin levels and the hepatic levels of glucokinase (a glycolytic enzyme) were significantly reduced in the T2DM rats, as compared to the control rats (Table 1). Fasting insulin levels were not significantly different between the control and control + SFFE-, or among the T2DM, T2DM + SFFE-, and T2DM + SFFE + brusatol-treated rats (Table 1). The control + SFFE- and T2DM + SFFE-treated rats exhibited lower levels of glucose and HBA1c, lower hepatic levels of G6P and FBP-1, and higher hepatic levels of glucokinase, as compared to their control counterparts (control or T2DM) (Table 1). However, the T2DM + SFFE + brusatol treatment did not show any significant variations in the levels of any of these markers, as compared with T2DM rats, indicating that the SFFE hypoglycemic effect is independent of modulating insulin levels but most likely due to an Nrf2-dependent suppression of hepatic gluconeogenesis.

### 3.4. SFFE Suppresses FFAs and Glycerol Release from Adipose Tissue in T2DM and Exerts a Hypolipidemic Effect in Both Control and T2DM Rats

The serum, hepatic, and stool TG and CHOL levels, as well as the serum and hepatic levels of FFA glycerol and the serum levels of LDL-c, were significantly higher in the T2DM rats, as compared to the control and control + SFFE-treated rats (Table 2). No significant changes in the serum glycerol levels or the stool levels of TGs and CHOL were observed when the control rats were compared with the control + SFFE-treated rats (Table 2). Glycerol levels were significantly higher in the T2DM rats, as compared with the control rats (Table 2). However, the serum levels of FFAs, as well as the serum and hepatic levels of TGs and CHOL, were significantly depleted in both the control + SFFE and T2DM + SFFE groups, as compared with the control rats or the T2DM rats, respectively (Table 2). Additionally, the T2DM + SFFE-treated rats had lower levels of glycerol, as compared with the T2DM model rats (Table 2). On the other hand, the T2DM + SFFE + brusatol-treated rats demonstrated a significant increase in the serum and hepatic levels of FFAs and glycerol, and they had significantly high serum and hepatic levels, as compared with the T2DM + SFFE-treated rats (Table 2). These values were not significantly different when these T2DM + SFFE + brusatol-treated rats were compared with the T2DM rats (Table 2). These data indicate that SFFE suppresses adipose tissue lipolysis only in T2DM, possibly by improving insulin sensitivity and action, and that it has a potent hypolipidemic effect that is independent of the excretion of lipids. However, both effects are Nrf2-dependent.

### 3.5. SFFE Improves Liver Function and Structure in T2DM Rats

**Figure 1:**
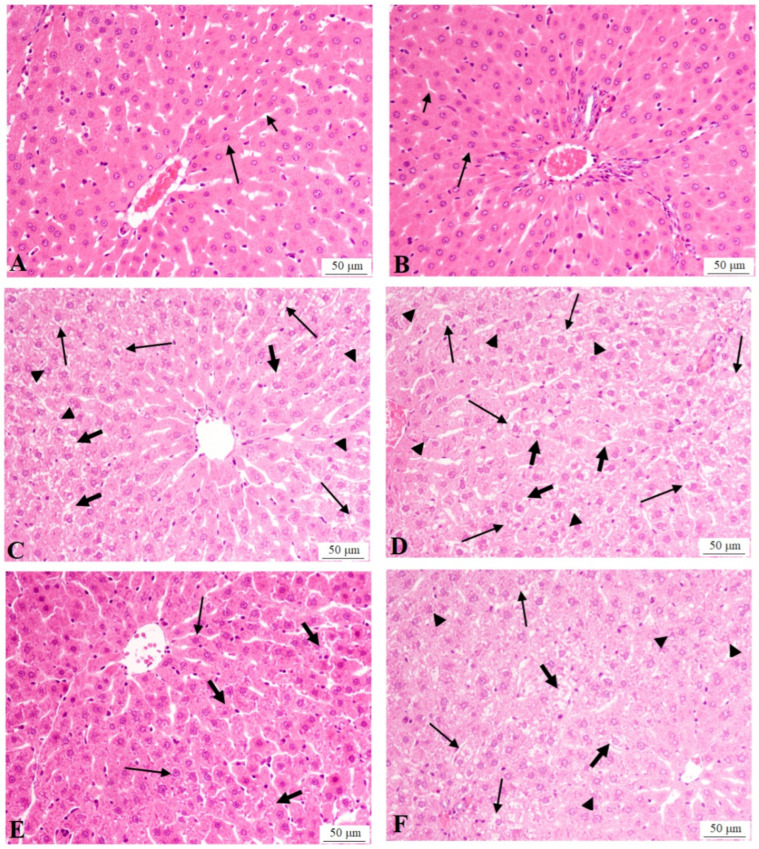
Histological sections from all groups of rats. (**A**,**B**) normal control and control + SFFE livers, showing normal hepatocyte structure with intact round nuclei (long arrow) and sinusoids (short arrow). (**C**,**D**) T2DM liver showing increased cytoplasmic vacuolization of large sizes (long arrows), damaged hepatocytes (thick short arrows), and pyknotic nuclei (arrow heads). (**E**) T2DM + SFFE liver showing almost-normal hepatocytes, with intact nuclei (long arrows) with very few cytoplasmic vacuoles in some hepatocytes of very small size (short arrows). (**F**) T2DM + SFFE + brusatol liver showing similar changes to those found in (**C**,**D**), including an increased number of damaged hepatocytes (thick short arrows), with large cytoplasmic vacuoles (long arrows) and pyknotic nuclei (arrowheads). H&E (200×).

The histological sections of the control and control + SFFE groups showed normal liver structures, with intact hepatocytes, central vein, and sinusoids (Figure 1). The serum levels of ALT, AST, and GTT were also not significantly varied between the control and control + SFFE groups (Table 3). Liver sections from the T2DM group showed increased hepatocyte damage, which is emphasized by the higher number of fat vacuoles and pyknotic nuclei, as well as increased levels of AST, ALT, and GTT, as compared with the control rats (Figure 1 and Table 3). On the contrary, the livers of the control + SFFE-treated rats showed almost-normal hepatocyte structures, with intact nuclei and almost-normal levels of all of the aforementioned enzymes, as compared with the T2DM model rats (Figure 1 and Table 3). The T2DM + SFFE + brusatol-treated rats showed similar pathological changes (damaged hepatocytes, increased vacuolization, and pyknotic nuclei) to those of the T2DM group (Figure 1). The serum levels of ALT, AST, and GTT were significantly higher in the T2DM + SFFE + brusatol-treated rats than in the T2DM + SFFE-treated rats (Table 3). No significant changes in the levels of ALT, AST, or GTT were observed between the T2DM rats and the T2DM + SFFE + brusatol-treated rats (Table 3). These data suggest that SFFE has Nrf2-dependent hepatic and hypolipidemic effects.

### 3.6. SFFE Increases the Cytoplasmic and Nuclear Transactivation of Nrf2 in the Livers of Control and T2DM Rats by Suppressing keap1 Transcription

**Figure 2:**
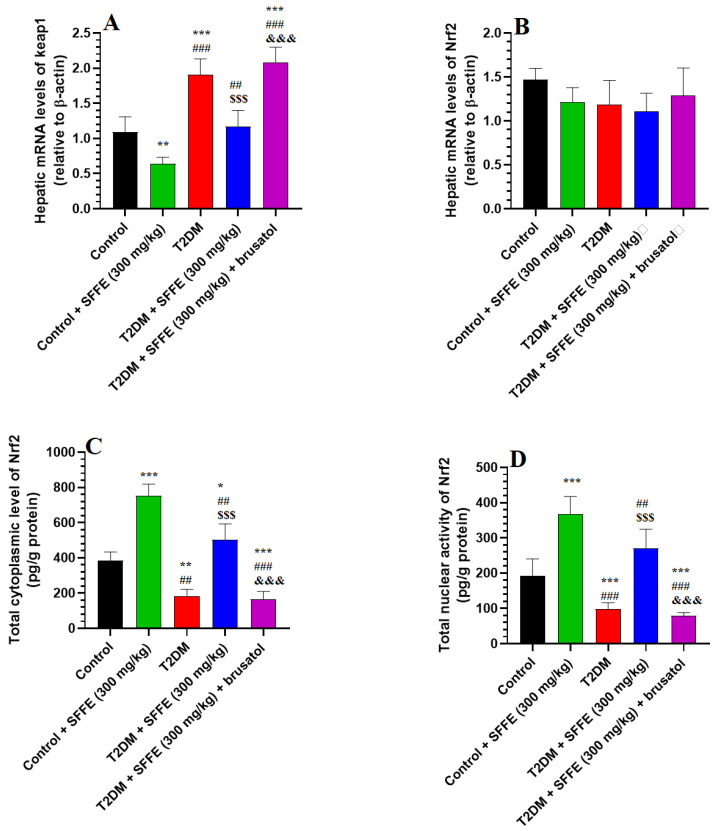
Transcriptional levels of keap1 (**A**) and Nrf2 (**B**), as well as cytoplasmic protein levels (**C**) and nuclear protein activity of Nrf2 (**D**) in the livers of all groups of rats. Data were analyzed using two-way ANOVA and Tukey’s post hoc *t*-test. Data are presented as mean ± SD of 8 rats/group. *, **, and ***: vs. control group, *p* < 0.05, 0.01, and 0.001; ^##^ and ^###^: vs. control + SFFE group, *p* < 0.01 and 0.001; ^$$$^: vs. T2DM group, *p* < 0.001; and ^&&&^: vs. T2DM + SFFE group, *p* < 0.001.

The mRNA levels of Nrf2 were not significantly different among the livers of different groups of rats (Figure 2B); the mRNA levels of keap1 were significantly increased but the cytoplasmic levels and nuclear activities of Nrf2 were significantly decreased in the livers of the T2DM rats, as compared to the control or control + T2DM rats (Figure 2A,C,D). The mRNA levels of keap1 were significantly decreased but the cytoplasmic levels and nuclear activities of Nrf2 were significantly increased in the livers of control + SFFE- and T2DM + SFFE-treated animals, as compared to the control and T2DM rats, respectively (Figure 2A,C,D). The levels of keap1 were significantly higher, and the cytoplasmic levels and nuclear activities of Nrf2 were not significantly lower, in the livers of the T2DM + SFFE + brusatol-treated rats, as compared to the T2DM + SFFE-treated rats (Figure 2A,C,D). No significant variations in the levels of any of these hepatic biochemical endpoints were observed between the T2DM rats and the T2DM + SFFE + brusatol-treated rats (Figure 2A,C,D). These data suggest that SFFE stimulates Nrf2 in the livers of rodents under both basal and diabetic conditions.

### 3.7. SFFE Reduces Lipid Peroxidations and Stimulates Antioxidant Expression in the Livers of the Control and T2DM Groups

**Figure 3:**
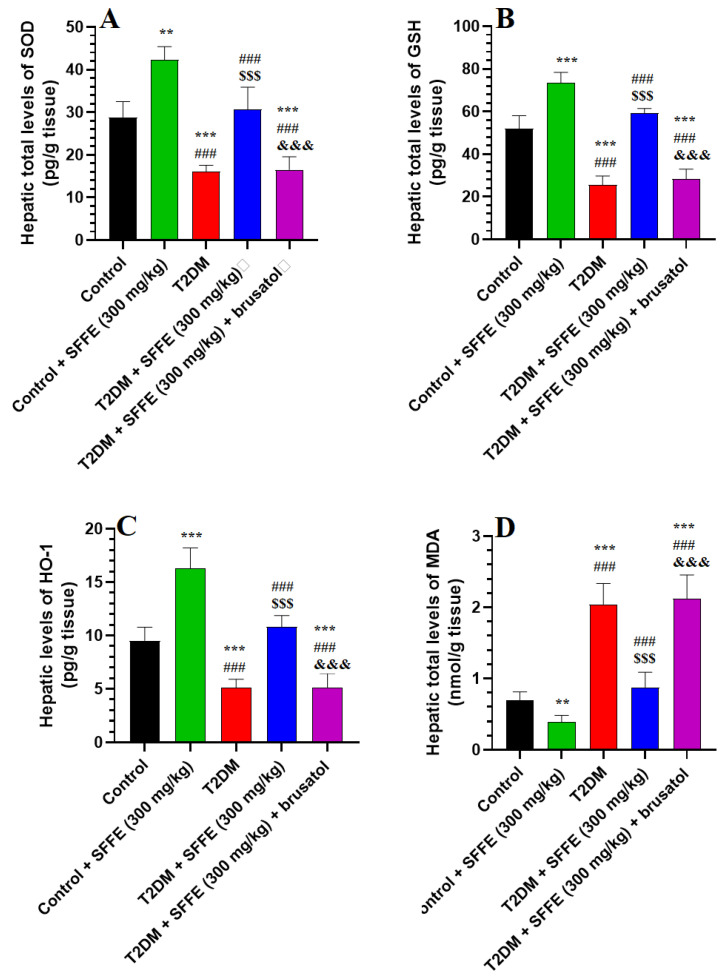
Total levels of superoxide dismutase (**A**), glutathione (GSH) (**B**), hemeoxygenase-1 (HO-1) (**C**), and malondialdehyde (MDA) (**D**) in the livers of all groups of rats. Data were analyzed using two-way ANOVA and Tukey’s post hoc *t*-test. Data are presented as mean ± SD of 8 rats/group. ** and ***: vs. control group, *p* < 0.01 and 0.001; and ^###^: vs. control + SFFE group, *p* < 0.001; ^$$$^: vs. T2DM group, *p* < 0.001; and ^&&&^: vs. T2DM + SFFE group, *p* < 0.001.

T2DM rats showed a significant reduction in their hepatic levels of GSH, SOD, and HO-1 and a significant increase in those of MDA, as compared to the control or control + SFFE-treated rats (Figure 3A–D). The hepatic levels of MDA were significantly reduced, while those of GSH, HO-1, and SOD were significantly increased, in the control + SFFE- and T2DM + SFFE-treated rats, as compared to their corresponding controls (i.e., control and T2DM rats, respectively) (Figure 3A–D). The livers of the T2DM + SFFE + brusatol-treated rats exhibited a significant increase in the levels of MDA, parallel with a significant decrease in the levels of GSH, SOD, and HO-1, as compared with the T2DM + SFFE-treated rats (Figure 3A–D). The levels of none these markers were significantly different, as compared to the T2DM rats. These data suggest that SFFE exhibits an antioxidant effect that involves Nrf2-dependent upregulation of GSH and antioxidant enzymes.

### 3.8. SFFE Surpasses NF-κB in the Livers of Both the Control and T2DM Rats

**Figure 4:**
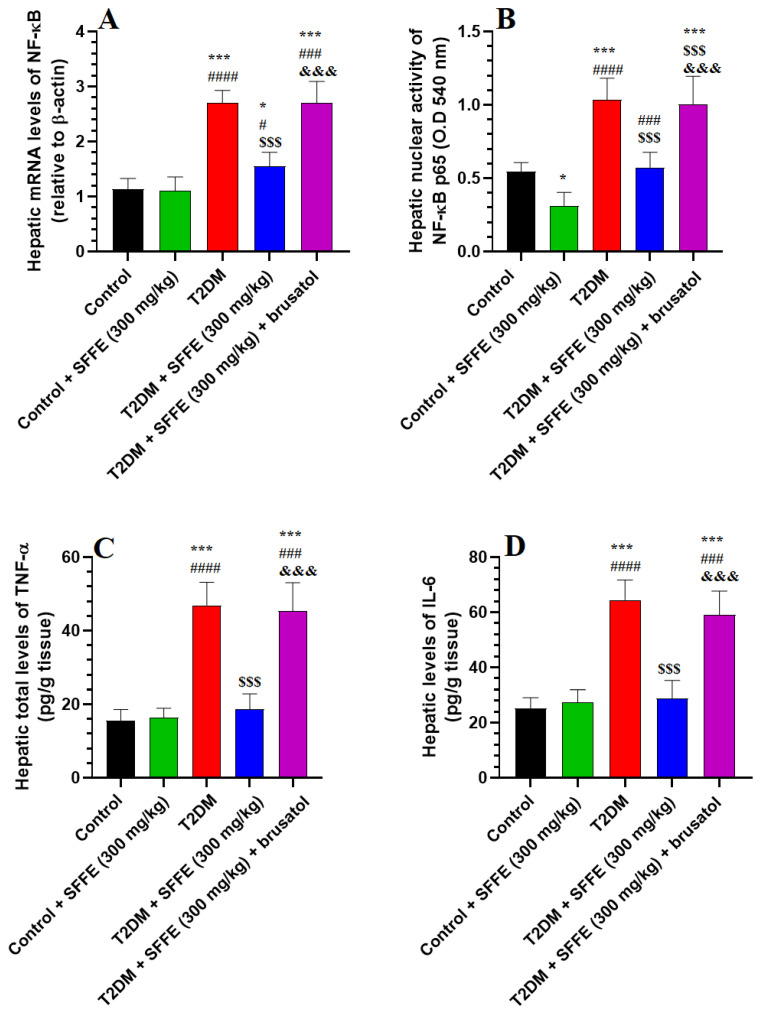
mRNA levels of NF-κB (**A**), nuclear activity of NF-κB (**B**), tumor necrosis factor-α (TNF-α) (**C**), and levels of interleukin-6 (**D**) in the livers of all groups of rats. Data were analyzed using two-way ANOVA and Tukey’s post hoc *t*-test. Data are presented as mean ± SD of 8 rats/group. * and ***: vs. control group, *p* < 0.05, 0.01, and 0.001; ^#^, ^###^ and ^####^: vs. control + SFFE group, *p* < 0.05, and 0.001, 0.0001; ^$$$^: vs. T2DM group, *p* < 0.001; and ^&&&^: vs. T2DM + SFFE group, *p* < 0.001.

SFFE-treated rats showed no significant changes in the hepatic mRNA levels of NF-κB, or the levels of IL-6 and TNF-α, as compared to control rats (Figure 4A,C,D). However, this group of rats showed significantly reduced nuclear activity of NF-κB, as compared to the control rats (Figure 4B). The livers of the T2DM rats showed significantly higher mRNA and nuclear activities of NF-κB levels, as well as TNF-α and IL-6 levels, as compared to those of the control or control + SFFE-treated rats (Figure 4A–D). This was reversed in the livers of the T2DM + SFFE + brusatol-treated rats (Figure 4A–D). However, the mRNA and nuclear activities of NF-κB, as well as the hepatic levels of IL-6 and TNF-α, were significantly higher in the T2DM + SFFE + brusatol-treated rats, as compared with T2DM + SFFE-treated rats (Figure 4A–D). No statistical differences were observed concerning the levels of any of these markers in the livers of T2DM + SFFE + brusatol-treated animals, as compared to the T2DM rats. These data suggest that SFFE exerts a potent anti-inflammatory effect in the livers of the control and T2DM groups, via Nrf2-dependent suppression of NF-κB.

### 3.9. SFFE Suppresses SREBP1, FAS, and ACC1 But Stimulates PPARα and CPT1 in the Livers of Control and T2DM Rats

**Figure 5:**
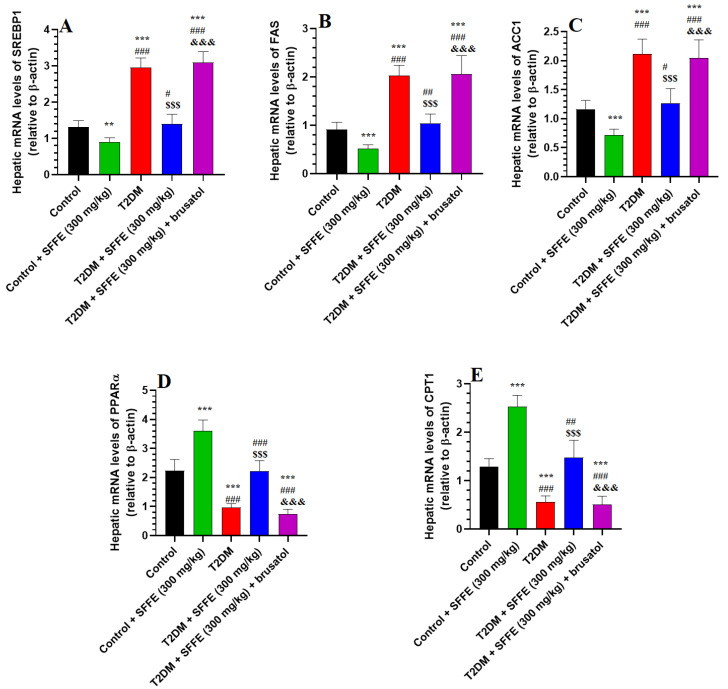
mRNA levels of SREBP1 (**A**), fatty acid synthase (**B**), acetyl CoA carboxylase (**C**) PPARα (**D**), and carnitine palmitoyltransferase 1 (CPT1) (**E**) in the livers of all groups of rats. Data were analyzed using two-way ANOVA and Tukey’s post hoc *t*-test. Data are presented as mean ± SD of 6 rats/group. ** and ***: vs. control group, *p* < 0.05 and 0.001; ^#^, ^##^, and ^###^: vs. control + SFFE group, *p* < 0.05, 0.1, and 0.001; ^$$$^: vs. T2DM group, *p* < 0.001; and ^&&&^: vs. T2DM + SFFE group, *p* < 0.001.

The livers of the T2DM rats showed significantly higher mRNA levels of SREBP1, FAS, and ACC-1 and lower mRNA levels of PPARα and CPT1 as compared to those of the control and control + SFFE-treated rats (Figure 5A–E). The livers of the control + SFFE and T2DM + SFFE groups showed lower mRNA levels of SREBP1, FAS, and ACC-1 and higher mRNA levels of PPARα and CPT1 when compared to the control and T2DM rats, respectively (Figure 5A–D). The mRNA levels of SREBP1, FAS, and ACC-1 were significantly increased, while the mRNA levels of PPARα and CPT1 were significantly reduced in the livers of the T2DM + SFFE + brusatol-treated rats, as compared to the T2DM + SFFE-treated rats (Figure 5A–E). No significant variations in the mRNA levels of any of these genes were observed between the T2DM and T2DM + SFFE + brusatol-treated rats (Figure 5A–E). These data indicate that SFFE can inhibit hepatic lipid accumulation via Nrf2-dependent suppression of hepatic de novo lipogenesis and stimulate FA oxidation by regulating key enzymes of these processes.

### 3.10. SFFE Suppresses Apoptosis in the Livers of T2DM Rats

The levels of Bax and caspase-3-were significantly increased, while the levels of Bcl2 were significantly reduced, in the livers of the T2DM rats, as compared to the control rats; these results were reversed in the livers of the T2DM + SFFE-treated rats (Table 4). No significant variations in the levels of Bax and caspase-3 were seen between the control and control + SFFE-treated rats (Table 4); however, levels of Bcl2 were significantly increased in the livers of the control + SFFE-treated rats, as compared to the control rats (Table 4). The livers of the T2DM + SFFE + brusatol-treated rats showed a significant increase in the levels of Bax and caspase-3 and a significant reduction in the levels of Bcl2 as compared to the T2DM rats (Table 4). No statistically significant changes in the levels of any of these markers were seen when the T2DM rats were compared with the T2DM + SFFE + brusatol-treated rats (Table 4). These data suggest that activation of Nrf2 enhances Bcl2 in the livers of the control and diabetic rats and suppresses Bax and caspase-3 activation in the livers of T2DM rats via an Nrf2-dependent mechanism.

## 4. Discussion

In this study, we demonstrate that safflower flower extract (SFFE) has significant protective effects against hepatic steatosis and damage in a rat model of type 2 diabetes mellitus (T2DM). SFFE’s protective role is mediated through hypoglycemic, hypolipidemic, antioxidant, and anti-inflammatory effects, primarily via modulation of endogenous antioxidants, SREBP1, PGC-1α, and NF-κB. Notably, the activation of the Nrf2 signaling pathway emerges as the key mechanism underlying these molecular effects. Inhibition of hepatic Nrf2 activation nullified SFFE’s protective benefits, leading to biochemical and pathological alterations akin to those in untreated T2DM rats.

We induced T2DM and hepatic steatosis in rats by administering a high-fat diet (HFD) for 4 weeks followed by a low-dose intraperitoneal injection of streptozotocin (STZ). This model, widely used for inducing T2DM and related multi-organ damage, mirrors human T2DM symptoms [17,64]. HFD leads to IR, dyslipidemia, and hyperglycemia, while STZ impairs insulin secretion, resulting in low insulin levels and progressive T2DM without external insulin administration [17,58,64]. The model exhibits increased food intake, reduced body weight, dyslipidemia, hepatic lipid deposition, and elevated markers of liver dysfunction [48,64,65,66], which SFFE treatment effectively mitigates. All of the aforementioned characteristics were observed in the T2DM rats in this study and were alleviated to almost-normal levels after treatment with SFFE, thus validating our animal model, as well as the hypoglycemic, hypolipidemic, hepatic anti-steatotic, and hepatic protective effects of this plant extract.

Insulin, an anabolic hormone, drives hepatic lipogenesis, glycogenesis, and glucose oxidation while inhibiting gluconeogenesis [67]. T2DM’s fasting hyperglycemia stems from elevated hepatic gluconeogenesis and impaired glucose uptake [68]. IR exacerbates hepatic glucose production and lipogenesis, leading to reduced body weight due to increased lipolysis and muscle wasting [69]. Key gluconeogenesis enzymes such as G-6-Pase and FBP-1 are upregulated, while glucose phosphorylation enzymes like hexokinase and glucokinase are downregulated in diabetic livers [58,70]. Previous research indicates that safflower oil and flower extracts have hypoglycemic effects in T1DM models, attributed to increased pancreatic beta-cell function and insulin secretion [45,46,47]. Safflower serotonin derivatives and other extracts can reduce fasting hyperglycemia by inhibiting intestinal α-glucosidase and improving insulin sensitivity [50,71,72]. Our study confirms that SFFE reduces fasting glucose levels in both T2DM and non-diabetic rats, with effects not linked to changes in food intake. The extract did not alter fasting insulin levels but did reduce serum FFAs and glycerol, suggesting improved peripheral insulin sensitivity without affecting insulin release from pancreatic cells. SFFE’s impact on hepatic gluconeogenesis and glucose oxidation is a novel finding. This effect may result from direct modulation of these pathways or indirect improvement in hepatic insulin sensitivity due to reduced lipid deposition, oxidative stress, and inflammation.

Hepatic steatosis, oxidative stress, and inflammation are hallmarks of T2DM [4,7]. Adipose tissue IR predicts oxidative damage and hepatic steatosis severity [73]. In T2DM, elevated lipolysis increases hepatic FFA influx, generating ROS and exacerbating inflammation and lipogenesis [74]. Major ROS sources in T2DM include mitochondrial and peroxisomal FA oxidation, mitochondrial dysfunction, ER stress, inflammatory cell activation, and depletion of antioxidants [11,75,76,77,78]. ROS-induced oxidative stress activates NF-κB and the mitochondrial apoptotic pathway, increasing Bax, cytochrome-c, caspases 3 and 9, and decreasing Bcl2 levels [10,79]. Our study observed similar oxidative stress, inflammation, and apoptosis in T2DM rat livers, evidenced by elevated liver function markers, lipid peroxides, reduced antioxidants, NF-κB activation, and altered Bax, caspase-3, and Bcl2 levels. SFFE treatment reversed these markers, suggesting antioxidant, anti-inflammatory, and anti-apoptotic effects. In vitro studies support SFFE’s antioxidant capabilities through ROS scavenging [80] and lipid peroxidation reduction [81,82]. Additionally, extracts from safflower leaves and seeds show hepatoprotective effects and improved antioxidant capacity [53,83,84].

Lipid metabolism in the liver involves the synthesis, export, and oxidation of FAs, regulated by transcription factors like SREBP1 and PPARα [85]. SREBP1 upregulates FA and TG synthesis and is activated in NAFLD due to high glucose levels, IR, and oxidative stress [86,87,88,89,90,91,92]. Conversely, PGC-1α and PPARα stimulate FA oxidation and mitochondrial biogenesis [93,94]. PGC-1α also suppresses NF-κB and upregulates mitochondrial antioxidants [95], while PPARα protects against oxidative damage by modulating inflammatory responses [96]. In NAFLD, SREBP1 activation and PGC-1α deficiency impair FA oxidation, making their modulation potential therapeutic targets [93,97]. SFFE’s hypolipidemic effects, including reduced hepatic lipid vacuolization and TG and cholesterol content, are likely due to suppression of de novo lipogenesis and stimulation of FA oxidation by regulating SREBP1, PPARα, and PGC-1α. Although prior studies reported hypolipidemic effects of safflower seeds and oil, this is the first study to show such molecular changes with SFFE in diabetic rats [45,46,47]. Previous research also highlights the role of safflower oil in increasing FA uptake and oxidation [50] and the impact of safflower seed extract on TG and cholesterol levels [48].

Nrf2 signaling activation is a potent strategy for treating NAFLD, with various plant phytochemicals demonstrating its effectiveness [13,14,34,98]. Nrf2, normally degraded by keap1, is activated under oxidative stress, leading to increased transcription of antioxidant genes and suppression of inflammation and apoptosis [16,99,100]. Nrf2 also enhances hepatic insulin signaling and reduces gluconeogenesis and lipogenesis [101]. Nrf2 activation promotes fatty acid oxidation and glucose uptake while inhibiting synthesis pathways [19,31,101,102,103,104,105,106,107]. In this study, SFFE treatment reduced glucose levels and reversed dyslipidemia through Nrf2 activation, as evidenced by the reversal of glucose and lipid levels and alterations in hepatic biomarkers. Brusatol, an Nrf2 inhibitor, abolished these effects, confirming Nrf2’s role in SFFE’s action.

Our findings indicate that SFFE activates Nrf2 and downregulates keap1, supporting its hypoglycemic, hypolipidemic, and hepatic protective effects. Prior studies have shown Nrf2 activation by safflower extracts in other contexts [54,108,109,110,111,112]. Future research should explore the specific compounds in SFFE responsible for Nrf2 activation and assess potential systemic effects beyond the liver.

In this study, we also conducted GC-MS analysis to provide a comprehensive profile of the diverse range of bioactive compounds present in the methanolic extract of safflower flowers. The compounds are listed in Table S1. The GC-MS analysis of the extract identified several bioactive compounds with potential therapeutic effects. Dihydroactinidiolide is notable for its antioxidant, antibacterial, anticancer, and neuroprotective properties [113,114,115], which may significantly contribute to the extract’s therapeutic potential. Caryophyllene oxide and palmitic acid are also prominent; the former has demonstrated anticancer activity [116,117,118,119], while the latter is known for its anti-inflammatory effects [120,121,122,123,124]. Additionally, linoleic acid supports the extract’s antimicrobial and antioxidant claims [125,126,127], and nonacosane may offer some antibacterial benefits [128,129]. Limonene dioxide exhibits antiproliferative impact and raises nitric oxide levels in lymphoma cells, and has neuroprotective properties [130,131]. Aromadendrene has antimicrobial properties by inhibiting *Staphylococcus aureus* and *Enterococcus faecalis* [132]. The combined effects of these compounds likely underpin the observed benefits of SFFE. Future research should focus on elucidating the interactions of these compounds to fully understand their therapeutic mechanisms.

In conclusion, SFFE exhibits significant hypoglycemic and hepatic protective effects through Nrf2 signaling pathway activation. Further studies are needed to identify the exact compounds involved and their effects on other tissues involved in glucose and lipid metabolism.

### Study Limitations and Future Prospectives

This study has several notable limitations and future prospects that warrant consideration. A key limitation is the lack of identification of specific bioactive compounds within the SFFE responsible for Nrf2 activation. While we demonstrated the efficacy of SFFE in mitigating hepatic steatosis and diabetes-related damage, the precise compounds contributing to these effects remain unidentified. Future research should focus on isolating and characterizing these active constituents to elucidate their individual or synergistic roles in the observed therapeutic outcomes. Additionally, our study primarily targeted hepatic metabolism, leaving a gap in understanding SFFE’s systemic effects on other organs, such as muscle and adipose tissue, which are crucial for comprehensive metabolic health. Future investigations should assess the impact of SFFE across multiple organ systems to capture its full therapeutic potential. Another limitation is the use of a rat model, which, while effective for simulating T2DM, may not fully replicate human pathophysiology. Therefore, translating these findings to human clinical settings will require cautious interpretation and validation through well-designed human trials. Furthermore, the detailed molecular mechanisms by which SFFE activates Nrf2 and interacts with other metabolic pathways were not fully explored. Future studies should delve deeper into these mechanisms to better understand the molecular basis of SFFE’s effects. Finally, long-term studies are needed to assess the chronic safety and efficacy of SFFE, including potential adverse effects and interactions with other treatments. Research exploring the synergistic effects of SFFE with other therapeutic strategies could also provide valuable insights into optimized treatment approaches for T2DM and hepatic conditions. Addressing these limitations and pursuing these avenues will enhance our understanding of SFFE’s potential and facilitate its application in clinical practice. Finally, in our study, one notable limitation is the lack of standardization of the methanolic extract of safflower flower (SFFE) to a specific active principle. While we conducted preliminary analyses to assess the composition of the extract and identify key bioactive compounds, we did not perform a rigorous standardization process. This means that while we confirmed the presence of several known bioactive constituents, the extract was not standardized to a single, specific active principle. Standardizing the extract to an active principle is crucial for ensuring consistent therapeutic effects and reproducibility of results. Future studies should address this limitation by implementing standardization protocols to validate and refine the extract’s efficacy, thereby enhancing the precision and applicability of our findings.

## Figures and Tables

**Table 1:** Changes in body and liver weights; plasma fasting glucose and insulin levels; and hepatic markers of glucose metabolism in all groups of rats.

Parameter	Control	Control + SFFE (300 mg/kg)	T2DM	T2DM + SFFE (300 mg/kg)	T2DM + SFFE (300 mg/kg) + Brusatol
Final body weight (g)	427.5 ± 34.6	436.4 ± 41.8	312.1 ± 25.6 ***^###^	418.3 ± 37.8 ^$$$^	301.2 ± 30.3 ***^###&&&^
Body weight gain (g)	315.3 ± 26.1	422.4 ± 37.4	219.1 ± 19.7 ***^###^	313.4 ± 32.5 ^$$$^	209.6 ± 18.5 ***^###&&&^
Liver weight (g)	16.5 ± 2.9	17.2 ± 3.1	25.6 ± 3.4 ***^###^	17.9 ± 2.5 ^$$$^	26.8 ± 2. ***^###&&&^
Liver index (%)	3.73 ± 0.66	3.87 ± 0.77	7.93 ± 1.73 ***^###^	4.33 ± 0.82 ^$$$^	8.79± 1.89 ***^###&&&^
Weekly food intake (last 4 weeks)	35.4 ± 2.4	33.6 ± 2.8	43.5 ± 3.1 ***^###^	34.8 ± 3.2 ***^###^	40.4 ± 2.7 ***^###^
FBG (mmol/L)	5.73 ± 0.68	4.56 ± 0.73 **	23.2 ± 2.83 ***^###^	7.73 ± 1.12 *^##$$$^	25.4 ± 3.63 ***^###&&&^
FBI (mU/L)	23.55 ± 2.89	21.24 ± 3.45	11.37 ± 1.82 ***^###^	12.78 ± 2.34 **^###^	11.89 ± 3.637 **^###^
HBA1c (%)	4.87 ± 0.89	4.63 ± 0.78	17.72 ± 1.49 ***^###^	6.73 ± 1.62 **^##$$$^	15.38 ± 1.62 ***^###&&&^
Hepatic glucokinase (pg/mg tissue)	12.27 ± 1.28	19.42 ± 1.79 ***	5.34 ± 0.69 ***^###^	11.45 ± 1.39 ^###$$$^	4.82 ± 0.55 ***^###&&&^
Hepatic G6Pase (U/mg tissue)	7.73 ± 0.92	4.37 ± 0.59 ***	33.25 ± 3.62 ***^###^	10.53 ± 1.48 *^###$$$^	30.5 ± 3.38 ***^###&&&^
Hepatic FBP-1 (pg/mg tissue)	84.3 ± 7.5	58.9 ± 6.7 ***	182.4 ± 15.9 ***^###^	93.4 ± 8.5 *^###$$$^	197.3 ± 20.5 ***^###&&&^

Data were analyzed using two-way ANOVA and Tukey’s post hoc *t*-test. Data are presented as mean ± SD of 8 rats/group. *, **, and ***: vs. control group, *p* < 0.05, 0.01, and 0.001; ^##^ and ^###^: vs. control + SFFE group, *p* < 0.01 and 0.001; ^$$$^: vs. T2DM group, *p* < 0.001; and ^&&&^: vs. T2DM + SFFE group, *p* < 0.001. Liver index was calculated as (liver weight/final body weight × 100). FBG: fasting blood glucose; FBI: fasting blood insulin; G6Pase: glucose-6-phosphatase; FBP-1: fructose bisphosphatase-1.

**Table 2:** Serum, hepatic, and fecal lipid profiles for all groups of rats.

	Parameter	Control	Control + SFFE (300 mg/kg)	T2DM	T2DM + SFFE (300 mg/kg)	T2DM + SFE (300 mg/kg) + Brusatol
Serum	TGs (mg/dL)	88.7 ± 6.8	71.3 ± 6.1 ***	212.2 ± 19.5 ***^###^	95.4 ± 8.6 *^##$$$^	205.3 ± 21.3 ***^###&&&^
	CHOL (mg/dL)	94.3 ± 8.9	77.45 ± 7.1 *	178.5 ± 15.8 ***^###^	101.2 ± 12.2 ^##$$$^	186.5 ± 17.9 ***^###&&&^
	LDL-c (mg/dL)	51.4 ± 5.3	42.2 ± 3.9 *	97.6 ± 10.5 ***^###^	55.4 ± 6.1 ^##$$$^	103.2 ± 9.4 ***^###&&&^
	FFAs (μmol/L)	412.9 ± 38.7	328.9 ± 33.5 **	905.3 ± 84.7 ***^###^	441.9 ± 36.5 ^###$$$^	975.4 ± 99.4 ***^###&&&^
	Glycerol (μmol/L)	66.5 ± 5.8	61.8 ± 6.9	142.2 ± 13.2 ***^###^	75.4 ± 6.4 ***^###^	153.2 ± 16.2 ****^###^
Liver	TG (mg/g tissue)	4.73 ± 0.56	3.98 ± 0.28 **	9.13 ± 0.83 ***^###^	0.512 ± 0.47 ^###$$$^	10.2 ± 1.3 ***^###&&&^
	CHOL (mg/g tissue)	2.43 ± 0.38	1.53 ± 0.25 **	5.73 ± 0.63 ***^###^	2.73 ± 0.36 ^###$$$^	5.83 ± 0.72 ***^###&&&^
Stool	CHOL (ng/dry g)	6.75 ± 0.72	6.13 ± 0.68	13.43 ± 1.8 ***^###^	11.72 ± 2.1 ***^###^	11.92 ± 1.82 ***^###^
	TG (ng/dry g)	2.49 ± 0.53	2.83 ± 0.51	7.82 ± 0.77 ***^###^	8.32 ± 0.69 ***^###^	8.41 ± 0.93 ***^###^

Data were analyzed using two-way ANOVA and Tukey’s post hoc *t*-test. Data are presented as mean ± SD of 8 rats/group. *, **, and ***: vs. control group, *p* < 0.05, 0.01, and 0.001; ^##^ and ^###^: vs. control + SFFE group, *p* < 0.01 and 0.001; ^$$$^: vs. T2DM group, *p* < 0.001; and ^&&&^: vs. T2DM + SFFE group, *p* < 0.001. TGs: total triglycerides; CHOL: total cholesterol; LDL-c: low-density lipoprotein cholesterol; and FFAs: free fatty acids.

**Table 3:** Changes in serum markers of liver function tests in all groups of rats.

Parameter	Control	Control + SFFE (300 mg/kg)	T2DM	T2DM + SFFE (300 mg/kg)	T2DM + SFFE (300 mg/kg) + Brusatol
AST (U/L)	33.5 ± 3.4	35.4 ± 3.7	75.4 ± 6.5 ***^###^	37.1 ± 4.7 ^$$$^	81.2 ± 8.6 ***^###&&&^
ALT (U/L)	22.9 ± 1.7	20.9 ± 2.1	54.8 ± 5.7 ***^###^	27.6 ± 2.6 *^#$$$^	51.2 ± 5.9 ***^###&&&^
GTT (U/L)	24.8 ± 2.8	25.6 ± 2.9	67.3 ± 5.8 ***^###^	26.5 ± 2.1 ^$$$^	63.4 ± 6.7 ***^###&&&^

Data were analyzed using two-way ANOVA and Tukey’s post hoc *t*-test. Data are presented as mean ± SD of 8 rats/group. * and ***: vs. control group, *p* < 0.05 and 0.001; ^#^ and ^###^: vs. control + SFFE group, *p* < 0.05 and 0.001; ^$$$^: vs. T2DM group, *p* < 0.001; and ^&&&^: vs. T2DM + SFFE group, *p* < 0.001. ALT: alanine aminotransferase (ALT) enzyme; GTT: gamma-glutamyl transpeptidase (GGT) enzyme; and AST: aspartate aminotransferase (AST) enzyme.

**Table 4:** Hepatic levels of some apoptotic and anti-apoptotic markers in all groups of rats.

Parameter	Control	Control + SFFE (300 mg/kg)	T2DM	T2DM + SFFE (300 mg/kg)	T2DM + SFFE (300 mg/kg) + Brusatol
Bax (pg/g tissue)	22.3 ± 2.5	24.3 ± 2.3	67.3 ± 5.4 ***^###^	28.5 ± 4.3 *^#$$$^	65.2 ± 6.8 ***^###&&&^
Bcl2 (nmol/g tissue)	42.3 ± 3.8	55.3 ± 6.3 **	19.4 ± 1.7 ***^###^	38.7 ± 3.6 ^$$$^	17.3 ± 2.2 ***^###&&&^
Caspaspe-3 (nmol/g tissue	8.4 ± 1.2	7.8 ± 1.1	27.4 ± 2.4 ***^###^	12.2 ± 1.4 *^##$$$^	26.8± 2.8 ***^###&&&^

Data were analyzed using two-way ANOVA and Tukey’s post hoc *t*-test. Data are presented as mean ± SD of 8 rats/group. *, ** and ***: vs. control group, *p* < 0.05, 0.01, and 0.001; ^#^, ^##^, and ^###^: vs. control + SFFE group, *p* < 0.05, 0.01, and 0.001; ^$$$^: vs. T2DM group, *p* < 0.001; and ^&&&^: vs. T2DM + SFFE group, *p* < 0.001.

## Data Availability

The datasets used and analyzed during the current study are available from the corresponding author upon reasonable request.

## Supplementary Materials

The following supporting information can be downloaded at: https://www.mdpi.com/article/10.3390/antiox13091098/s1, Figure S1: GC-MS chromatogram of the safflower flower methanol extract. Table S1: GC-MS profile of the methanol extract obtained from safflower flowers.
