# Supplementary material for: Carthamus tinctorius L. (Safflower) Flower Extract Attenuates Hepatic Injury and Steatosis in a Rat Model of Type 2 Diabetes Mellitus via Nrf2-Dependent Hypoglycemic, Antioxidant, and Hypolipidemic Effects
Source: Antioxidants (Basel). 2024 Sep 10;13(9):1098. doi: 10.3390/antiox13091098 (PMC11428842; doi:10.3390/antiox13091098)
Supplement: Supplementary file 1 [file antioxidants-13-01098-s001.zip › antioxidants-3108527-supplementary.pdf]

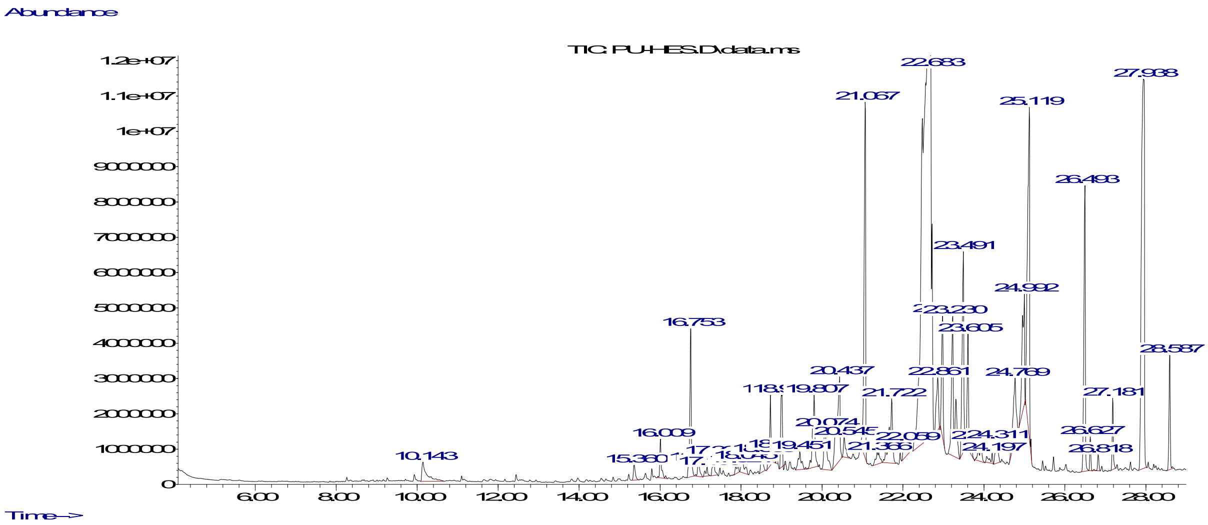

Figure S1: GC-MS chromatogram of the safflower flower methanol extract

Table S1: GC-MS profile of the methanol extract obtained from safflower flowers

**Compound Name**	**Biological activity**	**Chemical formula**	**Molecular weight (g/mol)**	**RT (min)**	**Area%**
2-(2-Butoxyethoxy)ethanol	Not reported	C8H18O3	162.23	10.143	0.907
Dihydroactinidiolide	It has antioxidant activity, antibacterial activity, anticancer activity and neuroprotective effects [64]. It also has acetylcholinesterase inhibitory, antioxidant and anti aggregation activities [114].	C11H16O2	180.24	15.367	0.29
Caryophyllene oxide	It possesses significant anticancer activities [117, 118]. It is platform for obtaining biologically active compounds [119].	C15H24O	220.35	16.009	0.60
Bicyclo[4.4.0]dec-1-ene, 2-isopropyl-5-methyl-9-methylene-	Not reported	C15H24	204.35	16.753	2.08
Aromadendrene	It has antimicrobial properties, by inhibiting Staphelococcus aureus and Ente rococcus faecalis [134].	C15H24	204.35	16.938	0.32
1(2H)-Naphthalenone, 3,4,4a,5,8,8a-hexahydro-8a-methyl-, trans-	Not reported	C11H16O	164.24	17.16	0.20

2(1H)-Benzocyclooctenone, decahydro-10a-methyl-, trans-	Not reported	C13H22O	194.31	17.326	0.38
Bicyclo[6.1.0]nonane, 9-(1-methylethylidene)-	Not reported	C12H20	164.29	17.924	0.22
1,8-Nonadiene, 2-methyl-5,7-dimethylene-	Not reported	C12H18	162.27	18.045	0.41
Calarene epoxide	Not reported	C15H24O	220.35	18.503	0.37
Arachidonic acid, trimethylsilyl ester	Not reported	C23H40O2Si	376.6	18.726	1.13
Limonene dioxide	It exhibits antiproliferative impact and raise nitric oxide levels in lymphoma cells, and has neuroprotective properties [132, 133].	C10H16O2	168.23	18.878	0.26
8-Nonen-2-one	Not reported	C9H16O	140.22	18.993	1.21
t-Butyl-dimethyl-[4-(2,6,6-trimethyl-cyclohex-2-enyl)-buta-1,3-dienyloxy]-silane	Not reported	C19H34OSi	306.6	19.451	0.55
1-(4-Bromobutyl)-2-piperidinone	Not reported	C9H16BrNO	234.13	19.807	1.73
9,10-Dimethyltricyclo[4.2.1.1(2,5)]decane-9,10-diol	Not reported	C12H20O2	196.29	20.074	1.26
Palmitic acid	It efficiently suppresses inflammation [122]. Palmitate, a methyl ester of palmitic acid induces cardiac hypertrophy and has a neuroprotective impact. It also reduces neuroinflammation and improves mitochondrial function [123,124].	C16H32O2	256.42	20.437	2.56
3beta-Acetoxy-19-hydroxyandrost-5-en-17-one	Not reported	C21H30O4	346.5	20.545	0.41
Hexadecanoic acid, trimethylsilyl ester	Not reported	C19H40O2Si	328.6	21.067	5.43
Cembrane	Cembrane-type diterpenes (0.45%) show anti-inflammatory effects [124].	C20H40	280.5	21.366	0.45
4-Acetoxy-1,2,3,5,6,7,8,8a-octahydroazulene	Not reported	C12H18O2	194.27	21.722	1.94
Linoleic acid	It has antibacterial, nematicidal and antioxidant properties [127].	C18H32O2	280.4	22.059	0.48
6-(3,5-Dimethyl-1H-pyrazol-1-yl)-3-methyl-1,2,4-triazolo[4,3-b][1,2,5,6]tetrazine	Not reported	C9H10N8	230.23	22.683	34.92

2,6-Dimethylocta-1,5,7-trien-3-ol	Not reported	C10H16O	152.23	22.861	1.13
4-Isopropylbenzyl bromide	Not reported	C10H13Br	213.11	22.975	1.78
Anhydrocumanin	Not reported	C15H20O3	248.32	23.491	3.35
Ethyl-alpha-[4-cyano-2-pyridyl]cyanoacetate	Not reported	C11H9N3O2	215.21	23.605	1.75
Ethyl levulinate	Not reported	C7H12O3	144.17	23.93	0.52
Diisooctyl adipate	Not reported	C22H42O4	370.6	24.197	0.26
Preg-4-en-3-one, 17.alpha.-hydroxy-17.beta.-cyano-	Not reported	C20H27NO2	313.4	24.311	0.59
Xanthinin	Not reported	C17H22O5	306.4	24.769	1.84
Cedran-diol, 8S,13-	Not reported	C15H26O2	238.37	24.992	2.65
8-Hydroxy-3,8a-dimethyl-5-methylene-2-oxododecahydronaphtho[2,3-b]furan-4-yl acetate	Not reported	C17H24O5	308.4	25.119	6.58
Octadecane	Not reported	C18H38	254.5	26.493	4.04
2-Pentacosanone	Not reported	C25H50O	366.7	26.627	0.34
Tert-butyl-dimethyl-tetracosoxysilane	Not reported	C30H64OSi	468.9	26.818	0.20
Octacosane	Not reported	C28H58	394.8	27.181	0.78
Nonacosane	It has little activity against *Escherichia coli* and *Bacillus subtilis*, *Pseudomonas aeruginosa* and *Staphylococcus aureus* [130].	C29H60	408.8	27.938	11.17
Triacontane	Not reported	C30H62	422.8	28.587	1.37
